# Clinical Manifestation and Phylogenetic Analysis of Peste des Petits Ruminants in Local Iraqi Breed Sheep in Al-Diwaniyah Province

**DOI:** 10.1155/2024/5579913

**Published:** 2024-08-06

**Authors:** Khalefa A. Mansour, Muthanna H. Hussain, Saad H. Al-Husseiny, Asaad J. Abid, Qassim H. Kshash

**Affiliations:** Department of Internal and Preventive Medicine College of Veterinary Medicine University of Al-Qadisiyah, Al-Diwaniyah 58001, Iraq

## Abstract

Peste des petits ruminants (PPR), a contagious virus that infects sheep and goats, damages livestock globally. This study examined the clinical features and phylogenetic analysis of the PPR virus in Iraqi breed sheep from Al-Diwaniyah province. A clinical trial of 610 sheep from different flocks found 150 oral lesions. Special primers for RT-PCR and Mega11 for phylogenetic analysis were used to study the PPR virus nucleoprotein (N) gene. The PPR infection rate was 44.6% in 4–12 month olds (*n* = 33/131) and 4.8% in 36–48 month olds (*n* = 3/75). A 608-bp PPR virus partial N gene sequence was found in 49.3% of samples by RT-PCR. In leucine, isoleucine, proline, glycine, alanine, glutamine, asparagine, threonine, serine, arginine, and lysine codons, 25 amino acid alterations were found. The protein codon 56 alanine-valine alteration was most significant. Moving from a smaller hydrophobic amino acid to one with a bigger side chain may reduce protein stability. Steric hindrance or protein shape change from Valine's extended side chain may impact folding, stability, functionality, and interactions with other molecules. Furthermore, phylogenetic analysis showed that the Nigerian strain (MN271586) was most similar to our Iraqi strain, with 100% identity and coverage. This study found the Peste des Petits Ruminants (PPR) virus in sheep flocks in Al-Diwaniyah Governorate, Iraq, which is genetically similar to neighboring countries. PPR virus strains must be monitored and genetically characterized since N gene alterations can affect infection and propagation.

## 1. Introduction

The PPR virus is known to trigger a highly contagious disease in small ruminants across Africa, the Middle East, Eurasia, and Asia [1]. PPR was first documented in Iraq in 1997 by [2] using serological assays. Furthermore, studies have been undertaken on Iran, Turkey, Saudi Arabia, and India [3–6]. PPR has numerous synonymous, including goat plague, kata, stomatitis-pneumoenteritis syndrome, and ovine rinderpest [7].

The International Committee on Virus Taxonomy identifies PPR as a member of the *Paramyxoviridae* family, subfamily *Paramyxovirinae*, genus *Morbillivirus*, and species caprinae. The genome is a negative-sense, single-stranded RNA. Morbilliviruses, including rinderpest and measles, are closely related and immunologically cross-reactive. The genome is a negative-sense, single-stranded RNA. Morbilliviruses, which include rinderpest and measles, are closely related and have immunological interactions. The nucleoprotein (N) gene, which produces the nucleocapsid when combined with the RNA genome, has been meticulously sequenced, revealing four lineages that correspond to the virus's geographical origins [8]. The nucleocapsid (N) attaches to the phosphoprotein (P) and the large protein (L), both of which crosslink during viral replication; the matrix protein (M) attaches it to the envelope; and the envelope contains hemagglutinin (H), which aids the virus's adhesion to host cells. The F fusion protein facilitates the infection of host cells. Neutralizing antibodies primarily regulate the orientation of the H protein [9].

Sheep and goats are the primary carriers of PPR, but cattle and pigs do not exhibit clinical signs or symptoms of the disease; therefore, they play an important role in virus transmission. PPR has been found in Laristan sheep, gazelles, Gumbok (Arabian, Arabian Mountain, Dorcas, Reem, Thompson), buffalo, impala, and Afghan goats [10]. Wildlife may serve as reservoirs for small ruminant pests, although there is little evidence that they sustain or disseminate them [11].

PPR infection in sheep and goat herds is expected to cause annual economic losses of US$1.44 billion, placing food security at risk in multiple countries. As a result, international organizations aspire to eliminate the disease by 2030 [12]. It is critical to act quickly to combat PPR in order to limit the large economic losses caused by the disease and reduce its spread, particularly in developing countries.

Clinical signs typically diagnose most PPR outbreaks, but it can be difficult to differentiate them from foot-and-mouth and bluetongue infections. As a result, PPRV diagnosis with antibodies is the most effective because infected animals have lifetime antibodies and a prolonged antibody response [13, 14]. RT-PCR is a highly sensitive and effective method for detecting morbilliviruses. This technique can extract genetic information from sequencing results in order to track the spread and migration of virus lineages [15]. Furthermore, in order to enhance the disease management program, it is necessary to conduct regular surveillance of both domestic and wild animals [16].

The following questions guided our study: how prevalent is PPR in Iraqi-bred sheep in Al-Diwaniyah province? What are the most notable clinical signs? Are clinical signs different in adjacent countries? What is the origin of the current stain in Iraq? What is a genetic variation? Does the mutation affect evaluation and phylogenetic analysis? And based on these questions, the aims of the present study were the evaluation of the clinical manifestations of Peste des Petits Ruminants (PPR) in Iraqi breed sheep in Al-Diwaniyah province, using PCR tests for confirmation of the PPR infection, investigating its molecular characteristics, and analyzing the phylogenetic relationships of PPR virus strains circulating in Iraqi breed sheep.

## 2. Materials and Methods

### 2.1. Samples

Clinical examination has done in a survey on 623 animals in which 150 samples of oral lesions were collected from several herds in Al-Qadisiyah governorate. Suspected animals ranged in 4–48 months old in both genders of local breeds of Iraqi sheep. The examination involved the general conditions, temperature, respiration, buccal cavity, eyes, and nostrils.

### 2.2. Viral RNA Extraction

By using a sterile scalpel, the oral lesions were collected gently from infected sheep. Then, the collected oral lesion samples were placed in a sterile microcentrifuge tube containing 200 *μ*l of Trizol solution. Moreover, a sterile pestle or homogenizer was used to disrupt the oral lesion samples in the Trizol solution. This step helps release the viral RNA from the cells and tissues into the Trizol solution. Then, the samples in Trizol solution were incubated at room temperature for a few minutes to allow for complete dissociation of nucleoprotein complexes. After incubation, the samples were stored at an appropriate temperature according to the Trizol manufacturer's instructions.

Follow the instructions of the Maxime™ RT-PCR PreMix (iNtRON Biotechnology, Korea), 200 *μ*l of suspension was loaded in 1.5 ml tubes, and 1 ml of AccuZol reagent was added. Then, 200 *μ*L of chloroform was added and shacked and incubated on ice for 5 minutes. These contents were centrifuged at 12000 rpm for 15 minutes at 4°C, and a fresh 1.5 ml tube was used to transfer the aqueous phase. Equal volume of isopropyl alcohol was added. After that, the contents of the tube were mixed well by turning it 4-5 times and then put in freeze −20°C for 10 min. Next, the supernatant was carefully removed after centrifuging at 12000 rpm for 10 min at 4°C. One milliliter of 80% ethanol was added, and the mixture was thoroughly vortexed. The supernatant carefully removed after centrifuging at 12000 rpm for 5 minutes at 40°C. The aqueous phase was removed, and the RNA pellet was left to dry for five minutes. Subsequently, the extracted RNA was then dissolved in diethyl pyrocarbonate (DEPC) water by running the solution through a pipette tip a few times and allowing it to rest in the water path at 55–60°C for 10 minutes. The RNA was stored at −70°C as well as PCR will be done. The concentration and purity of extracted RNA were measured using a nanodrop spectrophotometer at absorbance 260/280 nm at ratio 1.8 as pure RNA [17, 18].

### 2.3. Reverse Transcription PCR (RT-PCR)

#### 2.3.1. cDNA Synthesis Preparation

The complementary DNA synthesis master mix (with a total volume of 10 *μ*L) was prepared using 9 *μ*L of total RNA template and 1 *μ*L of Handom Hexamer primer (20 pmol) with M-MLV Reverse Transcriptase, following the instructions provided by iNtRON company, Korea. The components of the RT mix, including the mentioned ones, were added to the HiSenScriptTM RH(−) RT PreMixKit kit strip tubes, which contained all the necessary elements for cDNA synthesis. After this, the strip tubes underwent centrifugation at 3000 rpm for three minutes in an Exispin vortex centrifuge and were then incubated in a BioRad-USA thermocycler following a 2-step protocol: (step 1) cDNA synthesis (RT step) at 50°C for 1 hour, and (step 2) heat inactivation at 95°C for 5 minutes.

### 2.4. Preparing the PCR Master Mix

A 20 *μ*l total volume of the AccuPower® PreMix for PCR was utilized for the PCR reaction, following the manufacturer's instructions. This involved adding 5 *μ*l of cDNA template (5–50 ng), 1 *μ*l (10 pmol) each of the forward and reverse primer, and 13 *μ*l of PCR water. These PCR master mix components, along with other necessary components like Taq DNA polymerase, dNTPs, Tris-HCl pH: 9.0, KCl, MgCl2, stabilizer, and tracking dye, were added to the standard PCR PreMix Kit. The PCR tubes were then spun in an Exispin vortex centrifuge at 3000 rpm for three minutes before being placed in a PCR thermocycler. The RT-PCR primers (F: CCTTCTGGCCAAGGCTGTTA and R: CAGAGCTGACCTTTCCTGCA) designed to amplify a 608 bp segment of the nucleoprotein (N) gene for PPR detection were provided by Macrogen Company, Korea. The conventional PCR thermocycler settings used are listed in Table 1. Finally, the PCR products were visualized using a UV transilluminator and evaluated through agarose gel electrophoresis.

### 2.5. DNA Sequencing Method

The positive results of the morbillivirus N gene protein RT-PCR were sent to Macrogen Technology in Korea for DNA sequencing using the AB DNA sequencing system. The DNA sequencing analysis involved multiple alignment assessment using Clustal W alignment analysis and NCBI-BLAST to identify homologous sequences. Mutations and amino acid variations were analyzed using the bioEdit tool. Subsequently, MEGA software version 11 was used to perform phylogenetic analysis based on the sequences obtained from the current study and those available in GenBank.

### 2.6. Statistical Analysis

In the current study, we performed statistical tests to construct the tables and determine the link between clinical indications of PPR and its incidence across age cohorts. We calculated incidence rates and clinical indications using percentages (%). The authors used percentages to determine flock PPR and clinical sign frequency. Furthermore, we also used the *t*-test, correlation test (*r*), and *p* value (*P* < 0.05) for statistical analysis. We employed the *t*-test to show significant differences in means across unrelated groups and to determine the statistical significance of infection rates by age group. Furthermore, *t*-test has been used to assess incidence rates at 6–14 and 30–40 months. The correlation test coefficient (*r*) measured the strength and direction of the relationship between two variables. The authors used the correlation coefficient (*r*) to study clinical symptoms and infection rates.

## 3. Results

### 3.1. Clinical Signs and Observations

During clinical examination, suspected sheep with PPR showed symptoms such as high fever (above 40°C), diarrhea, oral ulcers, respiratory distress, serous discharge from the eyes and nostrils which later became purulent, as well as anorexia and depression. The most common and notable clinical sign was the presence of oral lesions. There were no significant differences in the infection rate between males 52.95% (323/610) and females 47.05% (287/610).

On the contrary, age was significant because there were 33/131 cases (25.2%) infected in the age 4–12 months, 16/91 cases (17.58%) infected in the age 24–36 months, and 22/326 cases (6.75%) infected in the age 12–24 months, as illustrated in a, b, and c in Table (2). Besides, it was not significant in age 36–48 months (d).

### 3.2. Correlation between Clinical Signs of PPR with Age Group

Based on Table 2, which shows PPR infection rates by age, Table 3 estimates the association between clinical symptoms of PPR and incidence and proportions for each age category. Four age groups exist: 4–12 months, 12–24 months, 24–36 months, and 36–48 months. Table 2 shows a significant decrease in popularity percentages between the 4–12-month group and the 36–48-month group, with resistance rates dropping to 4.8%. Various age groups display distinct signs of lymph node dysfunction. For instance, the age group of 6–14 months demonstrates elevated temperatures compared to the other age groups. Based on the data presented in Table 2, the symptoms of “oral ulcers” and “fever” rank as the most prevalent. All of the age groups depicted in the table exhibit these features.

As indicated by Table 3, between the ages of 4–12 months, there was a significant positive correlation (*r* = 0.93) between age and the incidence of mouth ulcers. High *t-*test results (10.87) and a very low *p* value (*p* < 0.0016) support this association and show a statistically significant relationship. There was a weak correlation (*r* = −0.03) between age and the incidence of mouth ulcers in the 12–24-month age group. A *p* value larger than >0.05 and a low *t*-test correlation coefficient (−0.40), which showed no discernible relationship, support this. There is a significant inverse relationship (*r* = −0.91) between age and the incidence of mouth ulcers within the 24–36-month age range. A statistically significant link is indicated by this association's enormous *t*-test result (−10.72) and incredibly low *p* value (*p* < 0.001). According to this study, as animals in this age group age, the incidence of mouth ulcers appears to be declining dramatically. Similarly, there is a considerable negative correlation (*r* = −0.87) between age and the incidence of mouth ulcers between the ages of 36–48 months. A statistically significant link is indicated by the huge *t*-test value (−8.86) and the incredibly low *p* value (*p* < 0.001). Within the specified age range, this investigation suggests a negative correlation between age and the incidence of mouth ulcers.

### 3.3. PCR Analysis

**Figure 1:**
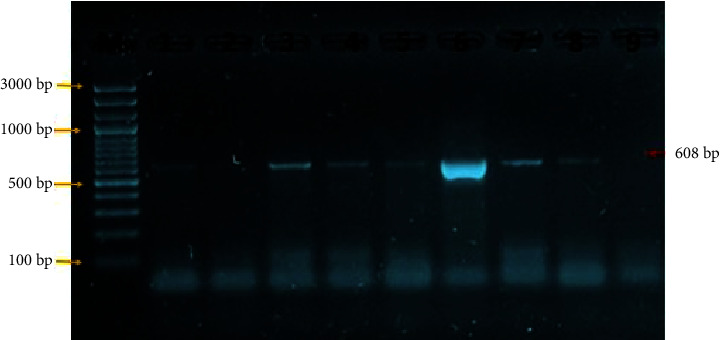
The results of agarose gel electrophoresis, showing the analysis of the RT-PCR products of the nucleocapsid protein (N) gene in Peste des petits ruminants' virus (PPR) oral lesion samples. Lane (M) represents the DNA marker ladder (3000−100 bp), and lanes (1, 2, 3, 4, 5, 6, 7, and 8) exhibit positive RT-PCR amplification of the PPR virus, resulting in a 608 bp PCR product size. However, lanes 1 and 9 show negative results.

The results of the RT-PCR assay for detection partial amplicon of the viral N gene of PPR–V of infected sheep in the province of Al-Qadisiyah 74/150 (49.3%) at (608 bp) as shown in Figure 1.

The result of this study revealed that the partial N gene sequences of the detected isolates in this investigation were related to those of PPR-V. These sequences have been deposited in the GenBank database with the accession codes (MK408669.1).

### 3.4. DNA Sequencing and Phylogenetic Analysis

**Figure 2:**
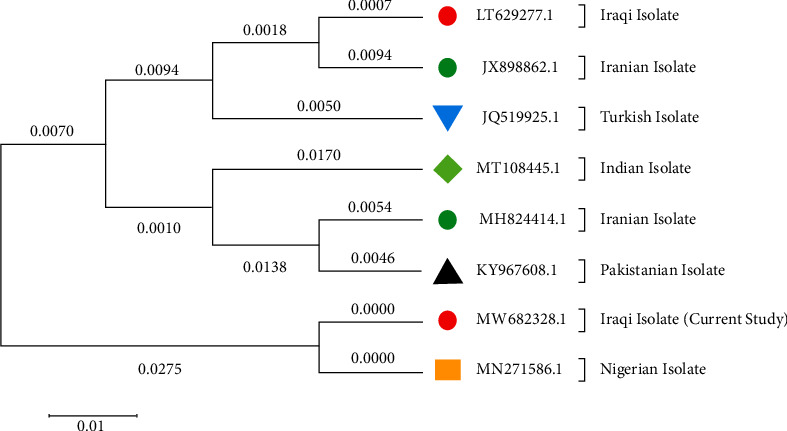
Shows the evolutionary phylogenetic tree of the Peste des petits ruminants' virus (PPR-V) N gene from the Iraqi isolate compared to the NCBI blast reference (strain/isolate). The phylogenetic tree demonstrates that the Iraqi isolates are grouped together with the Nigeria isolate (MN271586.1) and form a separate branch along with other strains (LT629277.1, JX898862.1, JO519925.1, MT108445.1, MH824414.1, and KY967609.1).

The NCBI-blast-related studies using the DNA sequencing method to identify genetic relationships revealed that the sequence of N gene was matched with obtainable sequenced using Mega 11. The sequencing obtained in this investigation was compared to 7 NCBI (strains/isolates) as mentioned in Table 4. After multiple sequence alignment, Mega 11 was used to create a phylogenetic tree (Figure 2) and percentage of homology between the N genes study sequence was estimated (Tables 5, 6, 7).

As demonstrated in Figure 2 and Table 5, the PPR virus detected in this study was highly related to those detected in Iran, Turkey, Erbil, and India in order which foretell the way disease was spread in and out of Iraq. Vaccination program should depend on the local strain rather than the imported vaccine because it will be more effective. Free movement of sheep and goat herds in the region is the most common cause beside the uncontrolled importation of animal product from Iran, Turkey, and India. Absence of the vaccine in the last decade is another important cause of the spread of the disease in the middle and the south of Iraq.

The findings of this study demonstrate that the Iraqi strain [MW682328] [19] exhibits 100% homology with Nigerian strain [MN271586], although it varies in composition when compared to Indian strains MT108445 and MT108445, turkey strain JQ51995, Iranian strains MH824414 and JX898862, and Pakistani strain KY967608. Moreover, the analysis of substitutions in the amino acid sequence of a protein encoded by the PPR virus genome, as presented in Tables 6 and 7, led to the classification of amino acids based on their structural and functional characteristics.

The amino acid analysis revealed 25 substitutions in the N gene of Peste des Petits ruminants' virus (PPR) at different positions (Table 7). For instance, substitution of hydrophobic amino acids like leucine (Leu) and isoleucine (Ile) influence the protein's interaction with hydrophobic regions, while proline (Pro) introduces structural kinks impacting flexibility. Additionally, small amino acids with small side chains like glycine (Gly) and alanine (Ala) may have similar roles in protein structure. Polar amino acids such as glutamine (Gln) and asparagine (Asn) affect protein function due to their ability to form hydrogen bonds, while threonine (Thr) and serine (Ser) influence hydrogen bonding in protein function through their hydroxyl groups. Positively charged amino acids like histidine (His), arginine (Arg), and lysine (Lys) are potentially involved in interactions with negatively charged molecules or enzyme catalysis, whereas negatively charged amino acids like glutamic acid (Glu) have distinct roles due to their negative charge.

Furthermore, the study identified a substitution between alanine (Ala) and valine (Val) at codon 56 of a protein sequence. This change from a smaller hydrophobic amino acid to a larger one with a bulkier side chain could significantly impact the protein's structure and stability. The larger side chain of valine might introduce steric hindrance or alter the local conformation of the protein, potentially affecting its overall fold or stability and subsequently its function and interactions with other molecules.

The Iraqi isolates were clustered in the same branch of the genetic tree with the isolates MN271586.1, KY967609.1, MH824414.1, and MTH108445.1. Also, the Iraqi isolates clustered with MN271586.1. The genetic tree also showed that the other isolates strains LT629277.1, JX898862.1, and JQ519925 were found to be clustered in a separate branch of the genetic tree as in Figure 2.

The results of nucleotide sequence alignments between the Nigeria isolate (MN271586.1) and the Iraqi isolate was (100%). In other hands, the results, as illustrated in Table 4, are obtainable that the present of homology ranged (95.93, 95.35, 95.02, 95.02, 95.02, and 96.02), respectively, with other global isolates (Iraq, Turkey, Iran, Pakistan, Iran, and India), respectively, obtained from NCBI with Iraqi isolates.

## 4. Discussion

This study found that sheep suspected of Peste des Petits ruminants (PPR) had high fever (above 40°C), diarrhea, oral ulcers, respiratory discomfort, purulent serous discharge from the eyes and nose, anorexia, and depression [20] which showed clinical and pathological abnormalities that indicate PPR. PPRV loves lung tissue but looks like rinderpest. A fever (pyrexia), eye and nose drainage, necrotizing and erosive mouth ulcers, gastrointestinal inflammation, diarrhea, and pneumonia are symptoms [21]. According to [22], the main clinical lesions in PPR infection were oral lesions. Furthermore, the authors in [23] reported that the most oral mucosa layer was congestion, sloughed, and ulcerated in infected goats.

Males 52.95% (323/610) and females 47.05% (287/610) exhibited similar infection rates, possibly due to the increased proportion of females in the herds. Age also played a significance, with 33 (25.2%) incidences in sheep aged 4–12 months and 3 (4.8%) in those aged 3-4 years. Some owners and herders claimed that males were more susceptible to PPRV; however, the authors in [24] discovered no gender difference. PCR identification of the N gene resulted in 49.3% infection rates (74/150 samples). Based on an investigation by [25], the nucleoprotein (N) gene was found in 63.2% and 89.1% of the samples, respectively, using conventional and reverse transcription real-time quantitative PCR. By comparing sequences from a brief part of the fusion (F) or nucleoprotein (N) gene, we concur with [10] molecular epidemiology of Peste des Petits ruminants' virus (PPRV). Moreover, Omer Babashekh et al. [26] found F and N genes in all suspected PPR-infected sheep using PCR.

The association between age and infection rate has several explanations: in younger animals with developing immune systems, mouth ulcers may be more common as immunological development continues. Second, vaccination status is important because younger animals may lack immunity due to inadequate vaccination or exposure, whereas older animals may have it through vaccination or natural exposure. Younger animals may be riskier or interact with sick people. At certain ages, specific clinical symptoms increase as the illness progresses. Finally, larger and more diversified populations are better able to estimate age-clinical sign correlations [27].

This study revealed a PPR virus that was closely linked to those reported in Iran, Turkey, Erbil, and India, showing its spread into and out of Iraq [25]. The study recommends using indigenous strains instead than imported vaccines for better efficacy. Free movement of sheep and goat herds and unrestrained imports of animal products from neighboring nations were said to spread the disease.

PPR N gene amino acid analysis revealed 25 changes. This substitution was based on structure-function similarities [28]. Hydrophobic Leu and Ile may affect protein interactions [29]. Protein chains kinked by proline changed shape and function [30]. Short side chains of glycine (Gly) and alanine (Ala) likely altered protein structure similarly [31]. Due to their polarity and hydrogen bond-forming ability, glutamine (Gln) and asparagine (Asn) may have had similar purposes. Hydroxyl group polarity of Thr and Ser implies hydrogen bonding [32]. Histidine, arginine, and lysine may have responded similarly due to their positive charges. Negatively charged glutamic acid (Glu) likely worked. Valine (Val) replaced alanine (Ala) at codon 56, creating a larger hydrophobic amino acid with a broader side chain. Protein stability and shape affect molecular interactions and function [33]. On the other hand, previous research identified fewer PPR virus N gene amino acid alterations [34].

In addition, the NCBI GenBank received amino acid sequences of positive PPR strains from wild and domestic goats, accession codes LT629276-LT629277 and LT882721-LT882728. PPR virus N gene amino acid changes were found in 10 locations in other research [25]. Ten positive strains from wild (B2, C1, L81, and N11) and domestic (P49, P56, S4, V67, V89, and V90) goats were also sent to NCBI GenBank for amino acid sequencing. These sequences have accession numbers LT629276-LT629277 and LT882721-LT882728.

The Iraqi strain [MW682328] was 100% comparable to the Nigerian strain [MN271586] but differed from the Indian strains MT108445 and MT108445, Turkish strain JQ51995, Iranian strains MH824414 and JX898862, and Pakistani strain KY967608 [25, 26, 35, 36]. The Peste des Petits ruminants' (PPR) viral genome amino acid sequence alterations were used to classify amino acids by structure and function. These sequences were also similar to strains, particularly the Nigerian strain [26, 37]. PPR infection epidemiology in this study was linked to eight strains from adjacent Asian and African nations [38, 39].

## 5. Conclusion

The current study indicates that the PPR symptoms in sheep included high fever, diarrhea, oral ulcers, respiratory pain, purulent serous discharge from the eyes and nose, anorexia, and sadness. Furthermore, the study found no significant differences in infection rates between males and females. However, age showed a significant difference in relation to infection rates. Moreover, oral ulcers were more prominent in 4–12-month age groups, where a strong *t*-test (10.87) and low *p* value (*p* < 0.0016) have been calculated. While we estimated a decrease in the prevalence of oral ulcer lesions in 24- to 36-month olds. These findings revealed that these animals' oral lesions exhibit an inverse relationship with age. A study of molecules and phylogeny showed that the PPR-V virus's N gene partial amplicon in Al-Qadisiyah sheep was 74/150 (49.3%) at 608 bp. The PPR N gene amino acid analysis revealed 25 substitutions. The analysis discovered a shift in protein codon 56 from Ala to Val. that may affect protein stability. Moreover, we recorded that the PPR strain of the local Iraqi sheep breed is identical 100% to the Nigerian strain and highly similar to the strains of neighboring countries. In conclusion, the present study indicated that PPR infection in the local Iraqi breed of sheep was genetically identical to that in neighboring countries. Since N gene mutations affect infection and dissemination, we must monitor and genetically define PPR virus strains.

## Figures and Tables

**Table 1:** PCR thermocycler program employed for PPR-virus diagnosis by PCR.

PCR step	Temp (°C)	Time	repeat
Initial denaturation	95	5 min	1

Denaturation	95	30 sec	30 cycle
Annealing	58	30 sec	
Extension	72	30 sec	

Final extension	72	5 min	1

**Table 2:** Infection rates of PPR-V according to age.

Age (months)	Inspected sheep	Infected sheep	Infection (%)
4–12	131	33	25.2^a^
12–24	326	22	6.75^c^
24–36	91	16	17.58^b^
36–48	62	3	4.8^d^

The superscript values ^a–d^ indicate statistical significance among the groups. Specifically, values with different superscripts (^a–d^) within the same column are significantly different from each other at *p* < 0.05.

**Table 3:** Correlation between clinical manifestations of Peste des Petits ruminants and the occurrence rate across different age groups.

Age (month)	Clinical signs	Clinical signs (%)	(*r*)	*t-*test	*p* value
4–12	Oral ulcers	30	0.93	10.87	<0.0016^*∗*^
	Fever	25			
	Diarrhoea	20			
	Respiratory distress	15			
	Anorexia	10			

12–24	Oral ulcers	28	−0.03	−0.40	>0.7111
	Fever	23			
	Diarrhoea	18			
	Respiratory distress	13			
	Anorexia	8			

24–36	Oral ulcers	25	−0.91	−10.72	<0.0001^*∗*^
	Fever	20			
	Diarrhoea	15			
	Respiratory distress	10			
	Anorexia	5			

36–48	Oral ulcers	4	−0.87	−8.86	<0.0001^*∗*^
	Fever	1			
	Diarrhoea	2			
	Respiratory distress	3			
	Anorexia	4			

^*∗* ^statistically significant.

**Table 4:** Accession numbers employed in this study to conduct the phylogenetic analysis.

No.	Accession no.	Source
1	MW682328	Current Study
2	MN271586	GenBank
3	LT629277	GenBank
4	JQ519925	GenBank
5	MH824414	GenBank
6	JX898862	GenBank
7	MT108445	GenBank
8	KY967608	GenBank

**Table 5:** Identity and coverage percentage of PPR virus sequences from different hosts and sources.

Sequence ID	Country	Host	Source	Identity (%)	Coverage
MN271586.1	Nigeria	Goat and sheep	Blood	100	100
LT629277.1	Iraq	Goat and sheep	Intestine	95.93	100
JQ519925.1	Turkey	Sheep and goats	Blood	95.35	100
MH824414.1	Iran	Capra hircus	Oral cavity	95.02	100
KY967609.1	Pakistan	Goat	Blood	95.02	100
JX898862.1	Iran	Goat	Lymphoid	95.02	100
MT108445.1	India	Caprine	Nasal swab	95.02	100

**Table 6:** A total of 52 places variations found in multiple sequence alignment of field *PPR* isolates from the current investigation (MW682328.1) and 8 GenBank nucleotide sequences of PPR samples.

No	SEQ	17	19	60	64	83	86	88	94	104	106	117	123	124	133	139	145	148	161	170	174	178	188	201	219	271
	CODONS	6	7	20	22	27	28	29	32	34	35	39	41	42	44	46	48	49	53	56	58	59	62	67	73	90
		CTA	GGG	ATA	ACC	GCC	CAC	CCC	CGA	AAG	GAG	GCG	CCC	CAA	CTG	AGA	AAA	GAA	CTC	GCC	AGA	GGG	CCG	CTG	GAT	CCG
1	MW682328	…	…	…	,,,	…	…	…	…	…	…	…	…	…	…	…	…	…	…	…	…	…	…	…	…	
2	MN271586	…	…	…	…	…	…	…	…	…	…	…	…	…	…	…	…	…	…	…	…	…	…	…	…	…
3	LT629277	…	…	ACA	AAC	GTC	CGC	CAC	CAA	…	GGG	…	…	…	CCG	AAA	ACA	…	CCC	GTC	…	GAG	CAG	…	…	…
4	JQ519925	…	GAG	ACA	AAC	GTC	CGC	CAC	CAA	…	GGG	…	…	…	CCG	AAA	ACA	…	CCC	GTC	…	GAG	CAG	…	…	…
5	MH824414	…	GAG	ACA	AAC	…	CGC	CAC	…	…	GGG	GGG	…	CGA	…	…	ACA	…	CCC	GTC	…	GAG	CGG	…	…	CTG
6	JX898862	…	GAG	ACA	AAC	GTC	CGC	CAC	CAA	AGG	GGG	…	…	…	CCG	AAA	ACA	GGA	…	…	…	GAG	CAG	…	…	…
7	MT108445	CCT	GAG	ACA	AAC	GTC	CGC	CAC	…	…	GGG	…	…	…	…	…	ACA	…	CCC	…	AAA	GAG	CGG	CCG	GGT	…
8	KY967608	…	GAG	ACA	AAC	…	CGC	CAC	,,,	…	GGG	…	CTC	CGA	CCG	…	ACA	…	CCC	GTC	…	GAG	CGG	…	…	CTG

**Table 7:** Amino acids substitution mutations recorded in the study isolate [MW682328] compared to 8 GenBank isolates of *PPR virus* samples.

No	SEQ	17	19	60	64	83	86	88	94	104	106	117	123	124	133	139	145	148	161	170	174	178	188	201	219	271
	CODONS	6	7	20	22	27	28	29	32	34	35	39	41	42	44	46	48	49	53	56	58	59	62	67	73	90
		leu	Gly	Ile	Thr	Ala	His	Pro	Arg	Lys	Glu	Ala	Pro	Gln	Leu	Arg	Lys	Glu	Leu	Ala	Arg	Gly	Pro	Leu	Asp	Pro
1	MW682328	…	…	…	,,,	…	…	…	…	…	…	…	…	…	…	…	…	…	…	…	…	…	…	…	…	
2	MN271586	…	…	…	…	…	…	…	…	…	…	…	…	…	…	…	…	…	…	…	…	…	…	…	…	…
3	LT629277	…	…	Thr	Asn	Val	Pro	His	Gln	…	Gly	…	…	…	Pro	Lys	Thr	…	Pro	Val	…	Glu	Gln	…	…	…
4	JQ519925	…	Glu	Thr	Asn	Val	Pro	His	Gln	…	Gly	…	…	…	Pro	Lys	Thr	…	Pro	Val	…	Glu	Gln	…	…	…
5	MH824414	…	Glu	Thr	Asn	…	Pro	His	…	…	Gly	Gly	…	Arg	…	…	Thr	…	Pro	Val	…	Glu	Gln	…	…	Leu
6	JX898862	…	Glu	Thr	Asn	Val	Pro	His	Gln	Arg	Gly	…	…	…	Pro	Lys	Thr	Gly	…	…	…	Glu	Gln	…	…	…
7	MT108445	Pro	Glu	Thr	Asn	Val	Pro	His	…	…	Gly	…	…	…	…	…	Thr	…	Pro	…	Lys	Glu	Gln	Pro	Gly	…
8	KY967608	…	Glu	Thr	Asn	…	Pro	His	,,,	…	Gly	…	Leu	Arg	Pro	…	Thr	…	Pro	Val	…	Glu	Gln	…	…	Leu

## Data Availability

The data sets used and analyzed during the current study are available from the corresponding author on reasonable request.

## References

[1] Donduashvili M., Goginashvili K., Toklikishvili N. (2018). Identification of peste des petits ruminants virus, Georgia, 2016. *Emerging Infectious Diseases*.

[2] Saoud H. (1997). Epidemiological and diagnostic study on some mixed infections of ruminants.

[3] Shahriari R., Khodakaram-Tafti A., Mohammadi A. (2019). Molecular characterization of peste des petits ruminants virus isolated from four outbreaks occurred in southern iran. *BMC Veterinary Research*.

[4] Şevik M., Sait A. (2015). Genetic characterization of peste des petits ruminants virus, turkey, 2009–2013. *Research in Veterinary Science*.

[5] Boshra H., Truong T., Babiuk S., Hemida M. G. (2015). Seroprevalence of sheep and goat pox, peste des petits ruminants and rift valley fever in saudi arabia. *PLoS One*.

[6] Balamurugan V., Varghese B., Muthuchelvan D. (2020). Seroprevalence of peste des petits ruminants in sheep and goats in eastern india. *Virusdisease*.

[7] Munir M., Zohari S., Berg M. (2012). *Molecular biology and pathogenesis of peste des petits ruminants virus*.

[8] Walker P. J., Siddell S. G., Lefkowitz E. J. (2021). Changes to virus taxonomy and to the international code of virus classification and nomenclature ratified by the international committee on taxonomy of viruses (2021). *Archives of Virology*.

[9] Baron M. D. (2014). The molecular biology of peste des petits ruminants virus. *Peste des Petits Ruminants Virus*.

[10] Banyard A. C., Parida S., Batten C., Oura C., Kwiatek O., Libeau G. (2010). Global distribution of peste des petits ruminants virus and prospects for improved diagnosis and control. *Journal of General Virology*.

[11] Tenuche O., Emikpe B., Godwin E., Enem S., Egwu G. (2023). Peste des petits ruminants: An update. *Microbiology Research Journal International*.

[12] Mariner J. C., Jones B. A., Rich K. M. (2016). The opportunity to eradicate peste des petits ruminants. *The Journal of Immunology*.

[13] Fentahun T., Woldie M. (2012). Reviewon peste des petits ruminants (ppr). *European Journal of Applied Sciences*.

[14] Ugochukwu I. C., Ezeasor C. K., Agina O. A. (2019). Peste des petits ruminants: Aetiology, pathology, immunology, disease status in africa, diagnosis, control, prevention and treatment: A review. *Notulae Scientia Biologicae*.

[15] Grant R. J., Banyard A. C., Barrett T., Saliki J. T., Romero C. H. (2009). Real-time rt-pcr assays for the rapid and differential detection of dolphin and porpoise morbilliviruses. *Journal of Virological Methods*.

[16] Prajapati M., Shrestha S. P., Kathayat D., Dou Y., Li Y., Zhang Z. (2021). Serological investigations of peste des petits ruminants in cattle of nepal. *Veterinary Medicine and Science*.

[17] Albayrak H., Alkan F. (2009). Ppr virus infection on sheep in blacksea region of Turkey: epidemiology and diagnosis by rt-pcr and virus isolation. *Veterinary Research Communications*.

[18] Amirouche A., Ait-Ali D., Nouri H. (2021). Trizol-based rna extraction for detection protocol for sars-cov-2 of coronavirus disease 2019. *New microbes and new infections*.

[19] Khalefa A. M., Muthanna H. H., Al-Husseiny S. H., Jassim A., Clinical Q. K. (2021). Molecular and Phylogeny Study of Peste des Petits Ruminants in Sheep of Iraq.

[20] Abera M. (2023). Review on pest des petits ruminants virus and its socioeconomic impact in small ruminants. *Journal ISSN*.

[21] Ishag O. M., Saeed I. K., Ali Y. H. (2015). Peste des petits ruminants outbreaks in white nile state, sudan: Research communication. *Onderstepoort Journal of Veterinary Research*.

[22] Toplu N. (2004). Characteristic and non-characteristic pathological findings in peste des petits ruminants (ppr) of sheep in the ege district of turkey. *Journal of Comparative Pathology*.

[23] Kumar P., Tripathi B., Sharma A. (2004). Pathological and immunohistochemical study of experimental peste des petits ruminants virus infection in goats. *Journal of Veterinary Medicine - Series B*.

[24] Hassan N. A. H., Suliman S. E. (2020). *Knowledge of pest de petites ruminants disease among owners of sheep and goats in sudan*.

[25] Khoran F. P., Candlan E. P., Hassan A. A., Isihak F. A., Abdulmawjood A., Khan I. U. (2021). Pheno-and genotypic characterization and identification of novel subtypes of peste des petits ruminants virus in domestic and captive wild goats in northern iraq. *BMC Microbiology*.

[26] Babashekh M., Rashid P. M. A., Marouf A. S., Raheem Z. H., Amin K. M. (2014). Genetic characterization of peste des petitis ruminants virus (pprv) from sulaimani/iraq by phylogenetic analysis and sequencing of nucleoprotein and fusion protein gene. *Journal of Zankoy Sulaimani-Part A*.

[27] Balamurugan V., Varghese B., Kumar K. V. (2020). Seroprevalence study of peste des petits ruminants in sheep and goats in the northern region of india. *August-2020*.

[28] Wu X., Liu F., Li L., Zou Y., Liu S., Wang Z. (2016). Major mutation events in structural genes of peste des petits ruminants virus through serial passages in vitro. *Virus Genes*.

[29] Deber C. M., Stone T. A. (2019). Relative role (s) of leucine versus isoleucine in the folding of membrane proteins. *Peptide Science*.

[30] Navaratnarajah C. K., Negi S., Braun W., Cattaneo R. (2012). Membrane fusion triggering: three modules with different structure and function in the upper half of the measles virus attachment protein stalk. *Journal of Biological Chemistry*.

[31] Op De Beeck A., Montserret R., Duvet S. (2000). The transmembrane domains of hepatitis c virus envelope glycoproteins e1 and e2 play a major role in heterodimerization. *Journal of Biological Chemistry*.

[32] Sarkar M., Saha S. (2020). Structural insight into the role of novel sars-cov-2 e protein: A potential target for vaccine development and other therapeutic strategies. *PLoS One*.

[33] Fan X., Kannan Villalan A., Hu Y., Wu X., Wang H., Wang X. (2024). Prediction of the potential host of peste des petits ruminants virus by the least common amino acid pattern in slam receptor. *Transboundary and Emerging Diseases*.

[34] Mishra A. R., Kumar Rath P., Kumar Panda S., Nayak D. (2020). Influence of mutation in nucleoprotein of peste-des-petits-ruminants virus (pprv) isolated from 2016 indian outbreak. *Small Ruminant Research*.

[35] Raoof H. S. (2023). Molecular characterization of circulating strains of the peste-des-petitis-ruminants virus in sulaimani province, Iraq. *Iraqi Journal of Veterinary Sciences*.

[36] Rudra P. G. (2019). *Prevalence and molecular characterization of peste des petits ruminants (ppr) in goat*.

[37] Mantip S., Sigismeau A., Shamaki D. (2022). Molecular epidemiology of peste des petits ruminants virus in nigeria: An update. *Transboundary and Emerging Diseases*.

[38] Pestil Z., Sait A., Ozbaser F. T., Bulut H. (2020). Molecular epidemiology of peste des petits ruminants cases associated with abortion in sheep and goat in marmara region of turkey, 2018. *Pakistan Veterinary Journal*.

[39] Pomeroy-Arthur U. E. (2023). *Morbillivirus Infections in Animal Hosts of the Serengeti District of Northern tanzania: Pprv and Cdv in Multi-Host Livestock Communities, and Cdv in African Wild Dogs (Lycaon Pictus)*.

